# Investigation on Perceptron Learning for Water Region Estimation Using Large-Scale Multispectral Images

**DOI:** 10.3390/s18124333

**Published:** 2018-12-07

**Authors:** Poliyapram Vinayaraj, Nevrez Imamoglu, Ryosuke Nakamura, Atsushi Oda

**Affiliations:** 1 AIST-Tokyo Tech Real World Big-Data Computation Open Innovation Laboratory (RWBC-OIL), Tokyo 152-8550, Japan; 2 National Institute of Advanced Industrial Technology (AIST), Tokyo 135-0064, Japan; nevrez.imamoglu@aist.go.jp (N.I.); r.nakamura@aist.go.jp (R.N.); x-oda@aist.go.jp (A.O.)

**Keywords:** AWEI, deep neural network, Landsat-8, MNDWI, PDWF, perceptron neural network, surface water bodies

## Abstract

Land cover classification and investigation of temporal changes are considered to be common applications of remote sensing. Water/non-water region estimation is one of the most fundamental classification tasks, analyzing the occurrence of water on the Earth’s surface. However, common remote sensing practices such as thresholding, spectral analysis, and statistical approaches are not sufficient to produce a globally adaptable water classification. The aim of this study is to develop a formula with automatically derived tuning parameters using perceptron neural networks for water/non-water region estimation, which we call the Perceptron-Derived Water Formula (PDWF), using Landsat-8 images. Water/non-water region estimates derived from PDWF were compared with three different approaches—Modified Normalized Difference Water Index (MNDWI), Automatic Water Extraction Index (AWEI), and Deep Convolutional Neural Network—using various case studies. Our proposed method outperforms all three approaches, showing a significant improvement in water/non-water region estimation. PDWF performance is consistently better even in cases of challenging conditions such as low reflectance due to hill shadows, building-shadows, and dark soils. Moreover, our study implemented a sunglint correction to adapt water/non-water region estimation over sunglint-affected pixels.

## 1. Introduction

The location and persistence of Earth’s surface water is changing due to various factors such as change in climate, seasons, and human activities. As Earth’s surface water is a vital resource, it is important to analyze its spatial and temporal changes. Accurate water/non-water region estimation is a key task for coastal change analysis, river channel change analysis [1], and shore/coast line extraction [2]. In addition, accurate water/non-water region estimation can be useful in estimating flood inundation and cloud masking. Therefore, accurate and automatic water/non-water region estimation is an important task. 

Several remote sensing image datasets are available that capture water bodies from around the globe, such as the Landsat mission and the Moderate Resolution Imaging Spectroradiometer (MODIS) [3,4,5]. Landsat-8 is a multispectral remote sensing sensor with 30 m spatial resolution and 16 days revisit capacity and is the latest in a continuous series of Landsat missions that began in 1972 [6], providing a vital resource to explore for surface water mapping. Water is a variable target with respect to its spectral reflectance at the wavelengths measured by the Landsat-8 images, which vary according to suspended sediments, chlorophyll concentration, dissolved organic matter [7], depth, and water bottom material [8]. Moreover, variables in satellite observation such as sun azimuth, sensor zenith, atmospheric condition, and geometry of the target will affect the image. At the global scale, all these conditions will be encountered at some point. Therefore, global water/non-water region estimation using remote sensing is challenging.

Several previous studies focused on water detection on a local scale [9,10] via water surface extraction using supervised classification methods [11], linear un-mixing [12], spectral indices, band thresholding, and other statistical approaches [3,13,14,15,16,17,18,19,20]. The most commonly applied spectral indices are the Normalized Difference Water Index (NDWI) [21] and the Modified Normalized Difference Water Index MNDWI (Xu, et al) [22], which utilize the reflectance of the green and Near-Infrared (NIR) bands and Shortwave Infrared (SWIR) bands. Band thresholding makes use of thresholds applied directly to the reflectance bands on derived spectral indices or on transformed bands [23,24]. Recently, Feyisa et al. [14] developed a water extraction index for maximizing the difference between water and non-water regions, and in most cases, zero was used as the threshold to delineate. Two different formulas were proposed by Feyisa et al. [14] for locations where shadows were the major source of accuracy loss and locations where shadows are not a major problem. These indices were claimed to have overcome the False Positives (FPs) induced by hill shadow or other low-reflectance surfaces such as dark soil and the False Negatives (FNs) mainly induced by sunglint and coastal turbidity. 

The key challenge when using existing simple water indices like NDWI and MNDWI is that the determination of the optimal threshold values that separate water and non-water pixels may vary with each location. More complex indices like Automatic Water Extraction Index (AWEI) also need to optimize the threshold regionally to obtain better results [14]. Therefore, the global adaptability or generality of the indices and their thresholds remains a major challenge for water/non-water region estimation tasks. 

In order to overcome these challenges, we propose a formula with perceptron-learning-derived parameters for water/non-water region estimation without a need for optimizing the threshold regionally. Our proposed Perceptron-Derived Water Formula (PDWF) is an extension of our previous work (Vinayaraj et al. [25]) in which the water extraction formula and parameter tuning used an iterative search process where the parameters with the best water/non-water region estimation results were selected. Nonetheless, our previous work was not able to distinguish hill shadows and building shadows or dark soils from non-water and needed additional data such as Digital Surface Model (DSM) [26] and Volcanic Soil Mask (VSM) [27] water/non-water region estimation. The PDWF aims to address these issues and to generate a formula which can effectively be used for water/non-water region estimation without any additional data apart from the Landsat-8 multispectral data. 

We also introduce an additional algorithm for sunglint correction as sunglint is a major cause of FNs in water/non-water region estimation. Sunglint is a location- and time-dependent phenomenon, which can affect many Landsat-8 images captured around the world, where the strength of the Specular Angle (SA) for each pixel should be estimated. However, none of the previous index-based water/non-water region estimation approaches have discussed the issue of sunglint-induced FNs. In addition, the occurrence of snow/ice also varies spatially and temporally; therefore, we adopt an algorithm to distinguish snow/ice from water. 

Recent developments in deep learning and Big Data analytics have also improved remote sensing image processing. Deep-learning-based data-driven approaches have recently been shown to have excellent performance in various satellite image analysis tasks such as feature extraction and region estimation [28,29], dimensionality reduction [30], and spatiotemporal feature learning [31]. Therefore, in addition to MNDWI and AWEI, we also compare our PDWF to the state-of-the-art deep learning network U-net [32] for water/non-water region estimation where we compare the accuracy of the results along with the time and resources needed to train the network.

The main contributions of this paper are as follows: We explored the efficacy of perceptron learning on global-scale Big Data for water/non-water region estimation using spectral features, and we compared it to current state-of-the-art models including deep learning approaches. A key difference from previous research is that the parameters were derived by automatic perceptron learning and the threshold is also determined automatically. Our developed PDWF can be used to estimate water/non-water regions from cloud-free, snow-free, sunglint-free Landsat-8 images. Hence, a combined simple decision-tree-based approach that includes PDWF, sunglint correction, and snow/ice correction can be used to estimate water/non-water regions from images with sunglint and snow cover. Several previous studies [3,15,16,27] heavily utilized additional datasets apart from Landsat-8 data such as DSM, VSM, glacier data, and urban data for improved water/non-water region estimation. Unlike previous studies, our proposed approach only uses Landsat-8 images and its supplementary information for water/non-water region estimation. Top of Atmosphere (TOA)-corrected Landsat-8 images were utilized; therefore, regional atmospheric correction was not necessary.

## 2. Materials and Methods 

**Figure 1:**
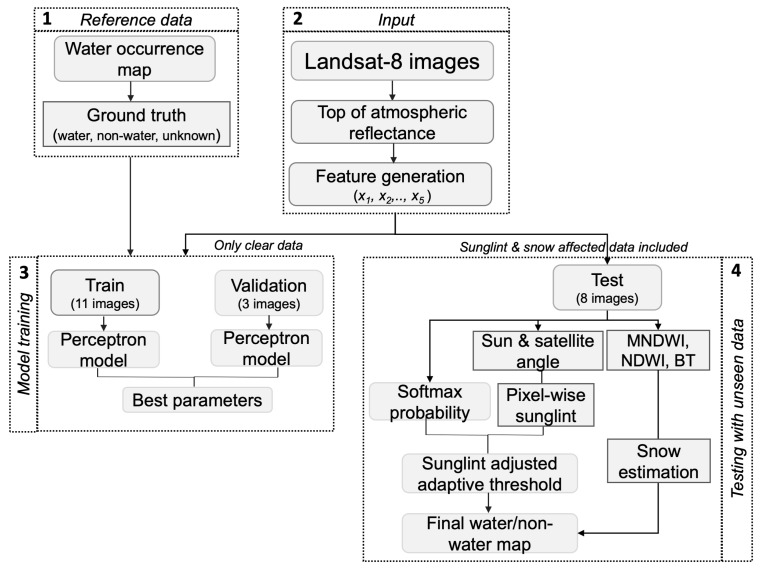
The workflow of proposed automatic water/non-water region estimation.

The workflow of our proposed approach is shown in Figure 1. The first step is reference data generation from Global Surface Water (GSW) (Pekel et al. [13]). Second, satellite images were preprocessed to generate TOA reflectance using the given rescaling coefficients. Third, a perceptron model was trained to derive the best parameters across 500 epochs. Fourth, the best parameters were used to estimate water/non-water regions for unseen test data. During training, only clear data were used, but our test data included satellite images which captured sunglint-affected or snow/ice-covered regions; hence, sunglint and snow/ice correction was performed.

### 2.1. Data Preparation

Geometrically and radiometrically corrected Landsat-8 images were transformed into TOA reflectance using the given scaling coefficients [33]. Details of the data are shown in Table 1. Twenty-two Landsat-8 images were collected over multiple locations around the globe, covering various surface water features such as coastal water, lakes, and rivers. Eleven cloud-free and snow/ice-free Landsat images were collected and used for training, an additional three were used for online validation, and eight were used for testing. Table 1 specifies the images which were affected by sunglint or captured from snow/ice regions.

Six bands from the visible and infrared spectral domain such as blue (0.452–0.512 μm), green (0.533–0.590 μm), red (0.636–0.673 μm), NIR (0.851–0.879 μm), SWIR1 (1.566–1.651 μm), and SWIR2 (2.107–2.294 μm) were used. Water occurrence data were collected from GSW (Pekel et al. [13]) for corresponding satellite images. A visual inspection was carried out for each GSW occurrence datapoint to determine a threshold to delineate water, non-water, and unknown. GSW shows the percentage of temporal water occurrence (0–100%), the pixels which were not able to be distinguished as water or non-water by visual inspection were considered as unknown data. Only water/non-water pixels (ground truth) were used for training the model and for validation. Data preparation was carried out in an Open Source Geographic Resources Analysis Support System (GRASS) Geographic Information System (GIS) [34].

The spectral reflectance of water decreases from shorter wavelengths to longer wavelengths due to the attenuation coefficient of water. Shorter-wavelength regions in the electromagnetic spectrum attenuate less when compared to longer wavelengths [35]. Relatively longer wavelengths such as NIR, SWIR1, and SWIR2 provide higher reflectance on most non-water regions such as vegetation, barren land, sandy beaches, and urban region but provide low reflectance over water regions. On the other hand, short wavelength bands, blue and green, generally provide low reflectance in non-water regions and high reflectance over water regions as compared to longer wavelengths [14]. We utilized this unique feature of the reflectance of water in different spectral bands and generated feature maps by subtracting longer-wavelength bands from shorter-wavelength bands. Three feature maps (*x*_1_, *x*_2_, and *x*_3_) were generated in order to maximize the difference between water and non-water, with positive values for water regions and negative values for non-water regions [14,25]. The first feature map (*x*_1_) was derived by subtracting the NIR band from the blue band, which shows higher values in water regions due to scattering in the blue band that keeps positive values even in deep-water regions. The second feature map (*x*_2_) was derived by subtracting the NIR band from the green band, which is mainly used to separate barren land, soil, and buildings from water. The third feature map (*x*_3_) was derived by subtracting the SWIR1 band from the red band, which separates vegetation, forest, and urban areas from water. The third feature map can also potentially separate turbid water and white caps due to wave-breaking from non-water regions. SWIR1 and SWIR2 were considered as the fourth (*x*_4_) and fifth (*x*_5_) features, respectively. These features were shown to be efficient for water/non-water region estimation in a previous study [25].

The aim of this study is to automatically derive parameters using perceptron learning for (*w*_1*i*_, …, *w*_5*i*_) and *b_i_* in Equation (1):

(1) $$S\left(x\right)=w_{1i}x_{1}\;+w_{2i}x_{2}\;+\;w_{3i}x_{3}\;+\;w_{4i}x_{4}\;+\;w_{5i}x_{5}\;+\;b_{i}$$

where *S* is the sum the dot products and *i* denotes the class among two classes: water and non-water. Different parameters for the water and non-water regions will be generated; hence, it will be easier to optimize the threshold value. 

### 2.2. Perceptron Learning

The perceptron is an intuitive easy to implement a machine learning algorithm [36]; it accepts an input vector *x* (*x*_1_, *x*_2_, ..., *x_n_*) as features and outputs either 1 or 0. The perceptron belongs to the category of supervised learning algorithms. The perceptron learns the weights for the input features in order to draw a linear decision boundary that allows us to discriminate between the two linearly separable regions. The workflow of the perceptron learning, illustrated in Figure 1, shows that two perceptions were used to derive the parameters for water and non-water regions. Five features were used. The first feature *x*_1_ denotes (blue − IR), the second feature *x*_2_ denotes (green − NIR), the third feature *x*_3_ denotes (red − SWIR1), the fourth feature *x*_4_ denotes (SWIR1), and the fifth feature x_5_ denotes (SWIR2). For each feature, the corresponding weights were randomly initialized, and a net function was used to sum the dot products of features and their corresponding weights as shown in Equation (2) [36]:

(2) $$S\left(x\right)\;=\;\overset{m}{\underset{n=1}{\sum }}w_{ni}\cdot x_{n}+b_{i}$$

(3) $$Z\left(x\right)=\;\{\begin{array}{c} 1\;\;\mathrm{if}\;\left(w_{ni}\cdot x_{n}+b_{i}\right)>0\\ 0\;\mathrm{otherwise} \end{array}$$

where *S* is the sum of the dot products, *m* is the total number of features, *w* is a randomly initialized weight that is updated at each iteration, *b* is the bias, *x* is the feature and *i* is the class and *Z* is the final classification. A single binary value was calculated for each pixel using Equation (3), and the softmax cross-entropy objective function was used along with Stochastic Gradient Descent (SGD) to update the weights during training. The weights which provided the highest accuracy were considered the optimal parameters.

#### 2.2.1. The Rectified Linear Unit (ReLU) Activation Function 

ReLU is a nonlinear activation function which assigns zero to negative values and grows linearly for positive values as shown in Equation (4) [37,38].

(4) R(x)=max(0,x)

**Figure 2:**
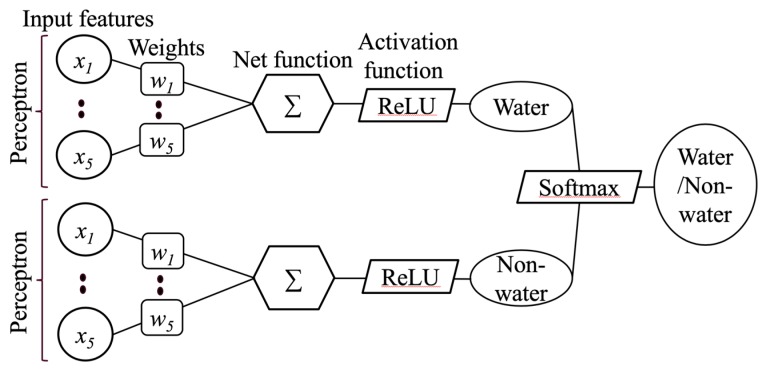
Perceptron neural network with two perceptrons: one for water region and another for non-water region.

Where, *R* is ReLU activation function, and it adds non-linearity in the neuron responses that can help to improve overall performance of the classification task. It does not saturate, which means that it is resistant to the vanishing gradient problem at least when *x* > 0, so the neurons do not backpropagate all zeros in at least half of their regions. ReLU activation was applied after weight summation as shown in Figure 2. 

#### 2.2.2. Softmax and Cross-entropy as the Loss Function

The softmax activation function [39,40] (Equation (5)) is used to get the binary probability maps from water and non-water features derived by each perceptron. The softmax function transforms the outputs of each neuron to be between 0 and 1. It divides each output such that the total sum of the outputs is equal to 1, as shown in Equation (5) [39,40]:

(5) $$Z\left(x\right)=\;\frac{e^{x_{wat}}}{e^{x_{wat}}+e^{x_{\text{non-wat}}}}$$

where *e* is the exponential function, *x_wat_* is the water class vector, *x_non-wat_* is the non-water class vector, and softmax calculates the probabilities for the *water* class. 

Cross-entropy is the loss function used to compare the output with the ground truth for updating the weights during backpropagation, as shown in Equation (6) [41]:

(6) $$H\left(p,q\right)=\;-\underset{x}{\sum }p\left(x\right)\;\log\;q\left(x\right)$$

where *p* is the ground truth labels [0,1], and *q* is the probabilities derived for each class by the softmax activation function. The cross-entropy value (*H*) was used to determine the weights to be updated. 

#### 2.2.3. Stochastic Gradient Descent (SGD) as the Optimizer

SGD is a stochastic approximation of the gradient descent optimization [42,43] which updates the weights of features in a perceptron at each iteration with limited batch size. Each parameter updated in SGD is calculated for every 100,000 pixels. Equation (7) [43] shows how the SGD weights are calculated:

(7) $$W=W-\alpha \nabla J\left(W,b,x^{\left(z\right)},y^{\left(z\right)}\right)$$

where *W* is the weight, α is the learning rate, ∇ is the gradient of the cost function *J*(*W*,*b*) with respect to changes in the weights, *x* and *y* are a training sample pair, and *z* is the number of samples in a batch. In this study, we used a learning rate of 0.001 and momentum of 0.09.

### 2.3. Algorithm to Reduce Sunglint-derived FNs

**Figure 3:**
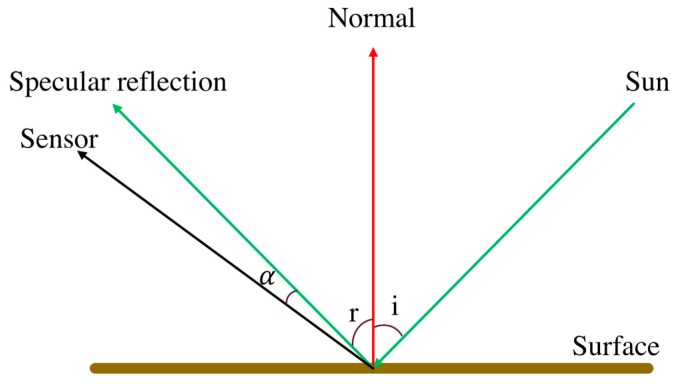
Schematic illustration of specular reflection and specular angle (α).

Sunglint occurs when sunlight reflects off the surface of water at the same angle that a satellite sensor observes it and is therefore a function of sea surface state, sun position, and viewing angle [44]. A smooth water surface reflects most of the incident light in the specular direction. Therefore, the appearance of its surface changes with viewing angle [45]. However, if the sea surface is relatively flat, a mirror-like reflection of the sun would be found at the horizontal specular point [46,47]. Figure 3 illustrates the phenomenon of specular reflection, where *i* is the incident angle and *r* is the reflectance angle. If *i* = *r*, the Specular Angle (*SA*) will be zero and strength of the reflected light is at its maximum. Water is a kind of illuminating surface and looks brighter, but its brightness will diminish as α becomes larger. Therefore, a low *SA* provides a brighter water surface region and may be estimated as non-water due to its high reflectance value. 

**Figure 4:**
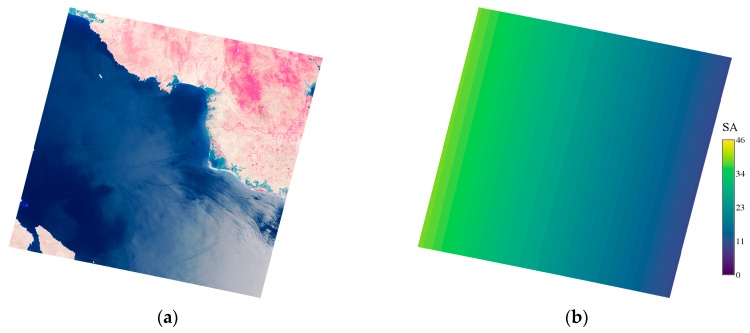
Sunglint-affected Landsat-8 image from the Gulf of California region: (**a**) the NIR, R, G composite clearly shows the sunglint-affected water region; (**b**) the derived specular angle (*SA*) for the corresponding region.

In the case of Landsat-8 in normal operation, the sensors view the earth at nadir Landsat [48]; therefore, the sun zenith angle can be a key factor that influences the specular angle. Hence, a lower sun zenith angle results in a greater chance for sunglint [49]. Even though the Landsat-8 is a nadir viewing satellite, pixelwise variation in the sensor zenith angle can be observed in the scene [44,45]. There is potential to vary the SA in the scene since the sensor zenith angle is linearly increasing from the center of the scene towards both sides [50,51]. Therefore, the SA also varies linearly and can be tracked using sun zenith, sensor zenith, sun azimuth, and sensor azimuth information. The sensor azimuth is calculated by using the orientation of the images with upper latitude, upper longitude, lower latitude, and lower longitude. The pixelwise sensor zenith was used along with the given parameters in the respective metadata file of the Landsat-8 image to generate pixelwise SA (Figure 4). Equation (8) [44,45,49] was used to calculate the *SA* and pixelwise *SA* as shown, and Equation (9) was used to correct the softmax-derived *Z* value. In Equation (9), *Z* will be added with the reciprocal value of the specular angle (SA); hence, the softmax-derived index values will be adjusted.

(8) $$SA=\;\cos^{-1}\{\cos\left(solar\;zenith\right)*\cos\left(sensor\;zenith\right)-\sin\left(solar\;zenith\right)\;*\sin\left(sensor\;zenith\right)*\cos\left(solar\;azimuth\;-\;sensor\;azimuth\right)\}$$

(9) $$SC=if\;SA<20,\left(Z+\;\frac{1}{SA}\right);\;if\;SA>35,(Z+\;\frac{1}{SA*3});\;else(Z+\;\frac{1}{SA*2})$$

In the equations above, *Z* is the softmax-derived index values, *SA* is the specular angle, and SC is the sunglint-corrected value for the softmax-derived index (0 to 1).

As a large SA results in less chance for sunglint, in order to make the sunglint correction negligible for bigger SA while keeping the smoothness of correction, we used an “if else” condition as shown in Equation (9). PDWF results in FNs in cases where *SA* is small, with stronger specular reflection, so the correction increases the value of the softmax-derived index according to the variation in *SA*. Therefore, water pixel values which were estimated as non-water regions due to a lower index value than the threshold (0.5) will be modified according to the *SA* and consequently estimated as water regions.

### 2.4. Algorithm to Distinguish Snow/Ice from Water Regions

The formula derived by perceptron learning is not able to distinguish between water and snow/ice because the model is trained only with snow-free and cloud-free images. Therefore, additional algorithms were adopted to distinguish snow from water. The MNDWI, NDWI, and Brightness Temperature (BT) were used (Equation (3)) (also see Appendix A). NDWI and MNDWI were computed using the equations (green − NIR)/(green + NIR) and (green − SWIR1)/(green + SWIR1), respectively [21,22]. Generally, the reflectance of NIR and SWIR show a linear relationship over most of the targets, but snow/ice shows high reflectance in the visible and near-infrared bands and low reflectance in shortwave infrared bands, leading to high MNDWI but low to moderate NDWI. A threshold (0.7) was used to reduce the confusion of water with snow/ice and a criterion of BT of <8 °C was also included to further distinguish snow from water [3]. The Landsat-8 images used in this study did not undergo atmospheric correction; therefore, in several images, the surface temperature of snow/ice can reach up to 8 °C. Equations (10) [3] and (11) show how to derive the final water/non-water region estimation using sunglint-corrected values.

(10) Snow = if (MNDWI > NDWI+0.7 && BT < 8,1,0)

(11) $$Final\;map\;=\;if\;(SC>0.5),\;water;if\;\left(SC\leq 0.5\right),non_{water};\;if\;\left(Snow==1\right),\text{non\_water}$$

## 3. Results and Discussion

In this study, five features (*x*_1_, *x*_2_, ..., *x_n_*) were used to estimate water/non-water regions using perceptron learning. Our previous research demonstrated [25] that these features could separate water and non-water using an appropriately fine-tuned threshold value. Hence, as many others have stated [4,14], the main challenge is to optimize the threshold and to determine the best combination of these features with appropriately tuned parameters. This study investigated perceptron learning to generate suitable parameters and thresholds. We carried out several experiments to investigate various aspects which affect the performance of the perceptron learning and performed several comparisons with training data noise, pretrained weights, random initialization, estimation using spectral bands, and estimation using features. An Open Source machine learning framework called Chainer [52] was used to train our models.

### 3.1. Five-fold Cross Model Training 

In order to derive the best parameters from the perceptron model, a five-fold cross model training approach was used. The data shown for train and validation in Table 1 were randomly shuffled and separated into five partitions which were used to train the model separately. Since the satellite images were not atmospherically corrected, there may be noise. As perceptron learning is similar to linear learning, the initialization of parameters is critical. Therefore, the five-fold cross model training was used to address this issue. Table 2 shows the training, validation, and testing accuracy. Evaluation of each fold was carried out to determine the best parameters in the training, validation, and testing phases. 

Experiments were carried out with random initialization of weights and initialization with pretrained weights. These pretrained weights were empirically assigned in our previous work [25] using a trial and error approach. In addition, six spectral bands were used to train the model instead of our features in order to compare the performance to spectral-band-based learning. Pretrained initialization of weights was not available in the case of spectral bands; hence, only random initialization was used in this case.

These experiments show that pretrained initialization for feature-based learning produces consistently better results. For random initialization, the feature-based and spectral-band-based experiments do not show consistent results. However, Folds 4 and 5 show the best accuracy using random initialization for spectral-band-based and feature-based approaches, respectively. Therefore, it is evident that initialization of weights was critical for perceptron learning, whereas random initialization is inconsistent. In contrast, experiments show that our features with pretrained weights (empirically assigned in our previous work [25]) produce consistently better results.

Fold 3 performs better in the training, validation, and testing phases with the feature-based pretrained learning approach. Hence, this study uses the best parameters derived from feature-based pretraining for Fold 3 for water/non-water estimation. The Fold 3 results were also compared with our previous work [25]: Fold 3 had an overall accuracy of 0.997, while our previous work’s accuracy was 0.994. It is expected that perceptron learning with 12 parameters may be affected by some unknown noise in the data or misleading random initialization. Therefore, five-fold cross model training was used to address this issue, and at least one of the folds can provide the best parameters. 

Data for training and validation were selected from cloud-free, snow/ice-free, and sunglint-free Landsat-8 images. Consequently, the automatically derived parameters are meant for water/non-region estimation in such cloud-free, snow-free, and sunglint-free Landsat-8 images. Two sets of parameters for Equation (1) were generated with appropriate weights and bias by the perceptron learning for water and non-water regions, as given below:

Parameters (water) = [0.989465, 1.14267147, 0.78721398, −0.93026412, −0.57805818], bias = [0.8181203];

Parameters (non-water) = [−1.04869103, −1.17793739, −0.73774189, 1.03303862, 0.65516961], bias = [0.88329011]. 

Here, features *x*_1_, *x*_2_, and *x*_3_ provide higher values for water pixels, while *x*_4_ and *x*_5_ provide lower values for water pixels compared to non-water pixels. Hence, perceptron learning utilizes this characteristic of the features to derive parameters in such a way that it maximizes the value for water pixels and minimizes the value of non-water pixels in the water layer and vice versa in the non-water layer (Figure 2). In the water layer, the parameters for *x*_1_, *x*_2_ and *x*_3_ are positive values while those for *x*_4_ and *x*_5_ are negative values; hence, in practice, *w_5i_x_5_* and *w_4i_x_4_* are subtracted from the sum of *w_1i_x_1_*, *w_2i_x_2_*, and *w_3i_x_3_*.

Further, a softmax activation function (Equation (4)) was used to automatically optimize the threshold and generate water/non-water estimation. Hence, there is no need to optimize the threshold independently. Five clear satellite images collected from various locations such as Lake Malawi, the Great Barrier Reef, Chilika Lake, Chiba, and Lake Victoria were used to evaluate the performance and generality of the developed formula. These five regions include various water types such as coastal, river, lake, sea, turbid water, etc., and non-water cover/use types such as vegetation, forest, urban, buildings, dark soils, hilly regions, shallow regions, etc. In clear datasets, the results were highly accurate with a very small number of erroneous pixels. However, another three sunglint-affected and snow/ice-covered images also were processed. As expected, the sunglint-affected regions produced a large number of FNs and the snow/ice regions produced a large number of FPs; these numbers were significantly reduced by the sunglint correction and snow/ice removal algorithms. 

### 3.2. Comparison with Other State-of-the-Art Methods

Several index-based approaches have been proposed by various studies [13,14]. Another method proposed by Xu et al. [22] (MNDWI) is one of the basic and simple band-ratio-based approaches. Therefore, we compared our results with those obtained using AWEI and MNDWI. In addition, we investigated the applicability or necessity of a deep learning model for water/non-water region estimation and implemented a deep learning model called U-net [32] for pixelwise water/non-water region estimation. In this study, the original U-net architecture was used with default hyperparameters [32] and zero padding to maintain the original image size. U-net consists of a contracting path (left side) with 10 convolution layers and an expansive path (right side) with 13 convolution layers. It concatenates lost features in the downsampling process with the appropriate features in the upsampling process. This particular aspect makes U-net an elegant deep learning model. 

The same number of Landsat-8 images as used for perceptron learning was used to train and validate the U-net (Table 1). Images were cropped into 45,567 samples with a size of 128 × 128 pixels and used to train the network. A total of 13,160 samples were used for on-line validation. Five-fold cross model training was also carried out for U-net model training. U-net shows consistently better accuracy in all folds for training, validation, and testing. Since U-net is a nonlinear deep learning model with millions of parameters, it was expected to produce consistently reasonable results in all the folds. 

**Figure 5:**
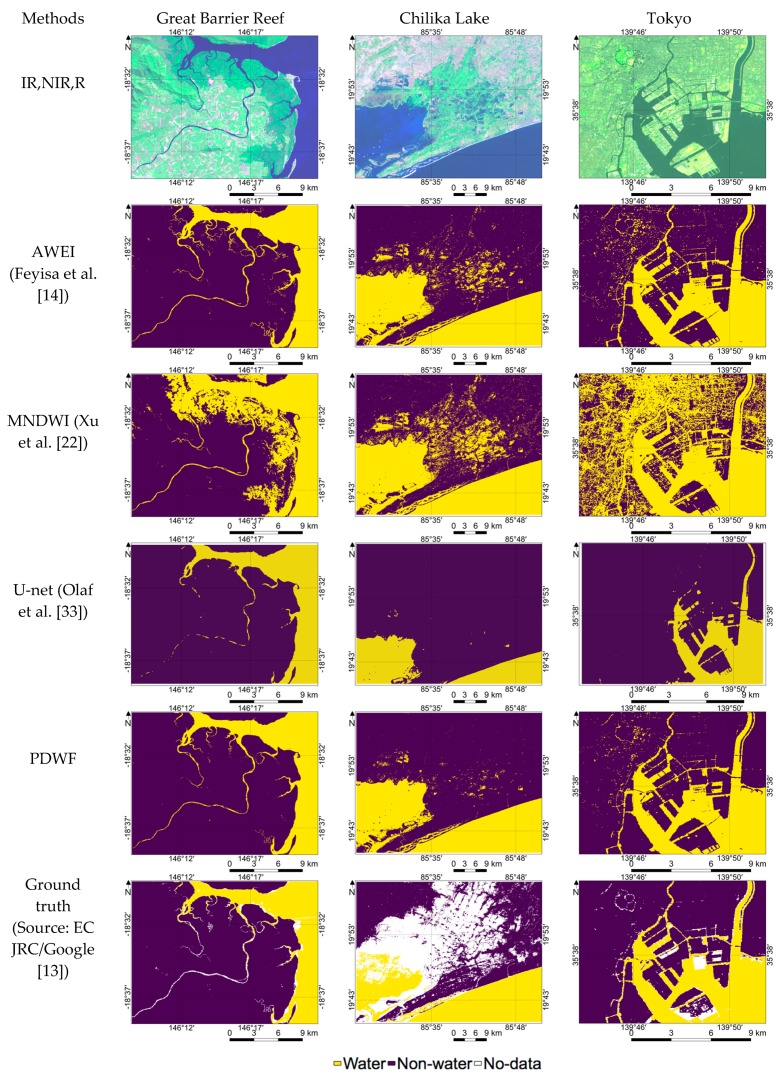
Detailed demonstration of water/non-water region estimation results derived from various methods over Great Barrier Reef, Chilika, and Chiba.

Our proposed method results were quantitatively and qualitatively compared with those from AWEI, MNDWI, and U-net. For clear data, five cloud-free, snow-free, and sunglint-free Landsat-8 images were used for testing. The results clearly showed that our proposed method outperforms AWEI in all of the five locations. In fact, AWEI is mainly proposed for Landsat TM images; however, there is no significant difference in the wavelength of spectral bands other than NIR with Landsat-8 images. As mentioned by Feyisa et al. [14], the original AWEI parameters were derived using atmospherically corrected images; hence, for a fair comparison with AWEI, atmospherically corrected surface reflectance data was used. It has been observed that our proposed method is highly efficient in eliminating FPs that result mainly from hill shadows, building shadows, dark soil, volcanic soil, etc., whereas AWEI is not able to eliminate FPs. For instance, Chiba gives high commission error in the AWEI method because of the FPs caused by building shadows. Figure 5 illustrates in detail the water/non-water region estimation in the Tokyo arearegion, which shows that the proposed method effectively estimated even building shadows as non-water whereas the AWEI method fails to do so. Figure 5 also shows that in the case of Chilika Lake, dark soils and wet paddy fields were estimated as water by AWEI.

MNDWI is the simplest and most basic approach for water/non-water region estimation using thresholds of zero. Pixels greater than zero were estimated as water and those less than zero as non-water regions. In all the case studies, our proposed method outperforms MNDWI which produces higher commission error mostly due to hill shadows, dark soils, building shadows, etc. In the case of Chiba, MNDWI produces high commission error (0.1123) due to the large FPs from the building shadows. Figure 5 demonstrates that MNDWI also struggles to correctly estimate hill shadow regions or dark soil and wet paddy fields in the Great Barrier Reef and Chilika Lake, respectively.

A deep learning model was implemented not only to compare the performance of the results but also to compare the resources, time for preparing the large amount of ground truth data, labelling, and time for prediction. U-net has a link between the left side and right side of the network which is used to concatenate lost features while downsampling. Therefore, U-net performs better than Deconvolution net (Noh et al. [53]) especially in medium-resolution satellite images. However, PDWF outperforms even U-net in all case studies expect for Lake Malawi because the study area has less complex water and land features. However, the average of the sum of the FPs and FNs shows that PDWF outperforms U-net overall (Table 3). In the case of the Great Barrier Reef, U-net outperforms all the other methods except for PDWF with low commission error (0.0003) and low omission error (0.0003). It shows relatively lower commission error compared to AWEI and MNDWI. In the case of Chilika, AWEI distinguishes low-lying paddy fields from water and therefore produces low commission error compared to U-net and MNDWI. In the case of Lake Victoria, U-net fails with high commission error due to wrongly estimating the vegetated shores of Lake Victoria as water.

In terms of qualitative evaluation of Figure 5, it is evident that U-net is efficient in reducing FPs induced by hill-shadows, dark soils, and paddy fields. However, U-net significantly fails to extract small or narrow water bodies such as ponds or river tributaries. In the case of the Great Barrier Reef, U-net was not efficient in estimating river tributaries. In the case of Chiba, U-net is worse at estimate small features compared to PDWF. This deficiency of U-net in medium-resolution-data water/non-water region estimation cannot be reflected in the quantitative evaluation due to the limitation of ground truth data available. Ground truth was also limited in the case of small or narrow water bodies, especially in regions of intersection between water and non-water.

**Figure 6:**
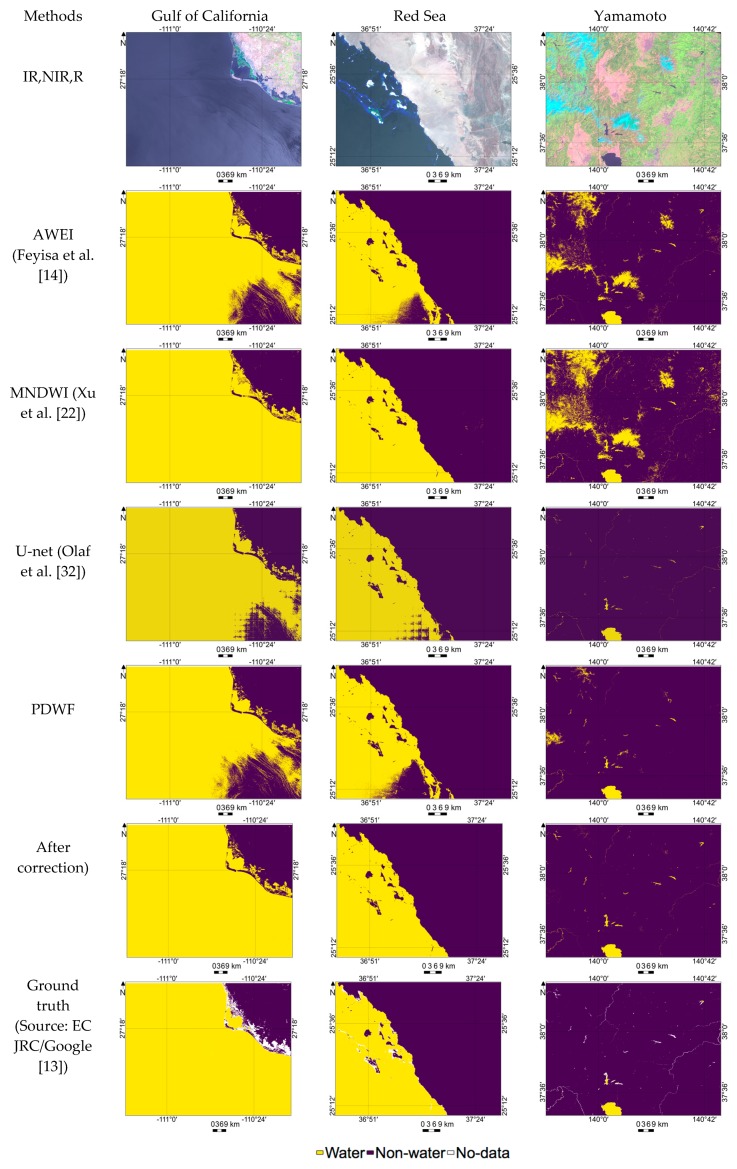
Detailed demonstration of water/non-water region estimation results derived from various methods on sunglint-affected and snow/ice-covered images.

PDWF shows consistently better performance in the case of clear images as expected, since the model was trained using clear images. This section evaluates the performance of PDWF in the case of sunglint-affected and snow/ice-covered regions. The Red Sea and Gulf of California image results shown in Table 4 were only for sunglint-affected images while the Yamamoto image was affected by sunglint and covered by snow/ice. Both quantitative and qualitative analysis show that AWEI and PDWF were impacted by the sunglint effect with high FNs, which is evident from the high omission error (Table 4). U-net also struggles with high FNs due to sunglint in the case of Red Sea and Gulf of California images. MNDWI performs comparatively better in the case of sunglint-affected regions with low omission error. Figure 6 also demonstrates that MNDWI performed better in sunglint-affected regions.

In the case of the Yamamoto image, snow/ice are significant factors that lead to erroneous estimation due to high FPs. In this case, MNDWI fails with high commission error (0.3613), and Figure 6 also shows that AWEI and MNDWI wrongly estimated snow/ice as water. PDWF performed better than AWEI and MNDWI with significantly lower commission error (0.0374). U-net performs best in this case, which shows that U-net is able to correctly estimate snow/ice as non-water regions.

#### Processing Time and Resource Comparison between PDWF and U-net 

For deep learning model, millions of parameters exist which utilize more resources and time for water/non-water region estimation using satellite image Big Data. Therefore, one of the main aims of this study is to use a limited number of parameters to reduce the resource and time cost for water/non-water region estimation. PDWF has 12 parameters while U-net has 7,766,915. Also, in the case of U-net prediction, the data have to be made into patches with 128 × 128 image size and projected back to the original shape after prediction. Table 5 shows separate columns for the time needed to generate patches and return them to their original shape. For PDWF, the time evaluation includes feature generation, and there is no need to generate patches. Separate testing over a CPU and a GPU were carried out to evaluate the processing time for each image, and the average of eight Landsat-8 images is shown in Table 5. An Intel(R) Xeon(R) E5-2630 v4 @ 2.20GHz CPU [54] with 10 cores (dual thread) and an NVIDIA Tesla P100 GPU computing processor (Tesla P100 SXM2 16 GB) [54] were used for the evaluation. For U-net, a batch size of 64 was used for both GPU and CPU because 64 was the maximum batch size which could be run on a single GPU. 

Table 5 shows that PDWF was much faster compared to U-net using either a CPU or a GPU. With a CPU, PDWF needed 2.66 s to finish the prediction of a single image, while U-net needed 1300.50 s to finish the same image, which was almost 500 times slower than PDWF. With a single GPU, PDWF needed only 0.06 s to complete the water/non-water estimation, while U-net needed 61.24 s, which was more than 750 times slower than PDWF. 

The performance of deep learning models may be improved either by tuning hyperparameters or functions such as activation functions, optimizer, loss function, or regulation, or by increasing the number of samples used for training. Also, deep learning models with more advanced architecture which can estimate small water regions from low-resolution images may be possible. However, these approaches require time and resources for labelling the training data and training the deep learning model and may increase the number of parameters, increasing the time for prediction. Therefore, a deep learning models may not be necessary for water/non-water region estimation from medium-resolution satellite images. Rather, simple and effective machine-learning-derived formulas may be a more reliable, time-effective, and resource-efficient solution. In all the clear data test sites used to evaluate the performance, PDWF consistently outperforms other methods, both index based and deep learning based. The goal of PDWF was to estimate water/non-water regions by minimizing the FPs; thus, the model was trained using images with low reflectance from hill shadows, building shadows, dark soils, and wet paddy fields. The model was not trained for sunglint-affected and snow-covered regions; hence, PDWF estimates sunglint as non-water regions and snow/ice as water regions. Previous studies [3,13,27] have used additional algorithms and data such as DEM for eliminating the FPs induced by hill shadows and building shadows. Apart from that, researchers have also manually created a VSM for the entire earth, which is highly time consuming and cannot identify newly formed volcanic soil areas. Therefore, PDWF was developed to significantly reduce FPs from these factors. Table 3 and Figure 5 clearly show the efficacy of PDWF in this task.

### 3.3. Improved Water/Non-Water Region Estimation after Sunglint and Snow/Ice Correction

As explained in Section 3.2 with Figure 6 and Table 4, Landsat-8 images which were severely affected by sunglint and snow/ice pixels were wrongly estimated with a large number of FPs and FNs, respectively. Images collected from the Gulf of California and the Red Sea were used to evaluate the performance of the proposed method, and further improvements were implemented using the sunglint correction algorithm given in Equations (8) and (9). An image from Yamamoto which includes snow/ice pixels and was also affected by sunglint was used to evaluate the performance of the proposed method along with sunglint correction and snow/ice correction.

High omission error in sunglint-affected regions and high commission error in the snow/ice regions were expected, as shown in Table 6. For the Gulf of California and the Red Sea, the accuracies significantly increased from 0.9167 to 0.9994 and from 0.9823 to 0.9987, respectively. Figure 6 shows the result after sunglint and snow/ice correction, with significant improvement after corrections in all the cases. The improvement in the accuracy was from a significant reduction in omission error due to sunglint correction (Table 6). 

In the case of Yamamoto, sunglint correction slightly reduced the sum of FPs and FNs from 269,177 to 268,633 by lowering the omission error. Snow/ice correction significantly reduced the sum of FPs and FNs from 268,633 to 62,666 by lowering the commission error. Generally, a simple decision tree made by combining PDWF with sunglint correction and snow/ice correction is a feasible approach for water/non-water region estimation. As explained in Section 2.3, when the *SA* was low, the specular reflectance was higher; therefore, the sunglint effect was increased. For the Gulf of California and the Red Sea, the pixelwise *SA* ranges from 9.5° to 39° and from 6.7° to 36.5°, respectively; therefore, the sunglint was significant. For Yamamoto, the pixelwise *SA* ranged from 20° to 41°; therefore, sunglint was not significant and the accuracy increase from sunglint correction was consequently not significant. Nonetheless, FPs induced by snow/ice were a major source of error.

### 3.4. Performance Evaluation in Detecting Smaller Water and Non-water Features

Detecting smaller water regions, such as narrow streams, and accurate delineation of water and land were also considered a challenge in water/non-water estimation. Here we demonstrate the feasibility of the proposed model in these scenarios. Several examples of extracting smaller water regions have been observed in our global-scale survey using the proposed method. 

**Figure 7:**
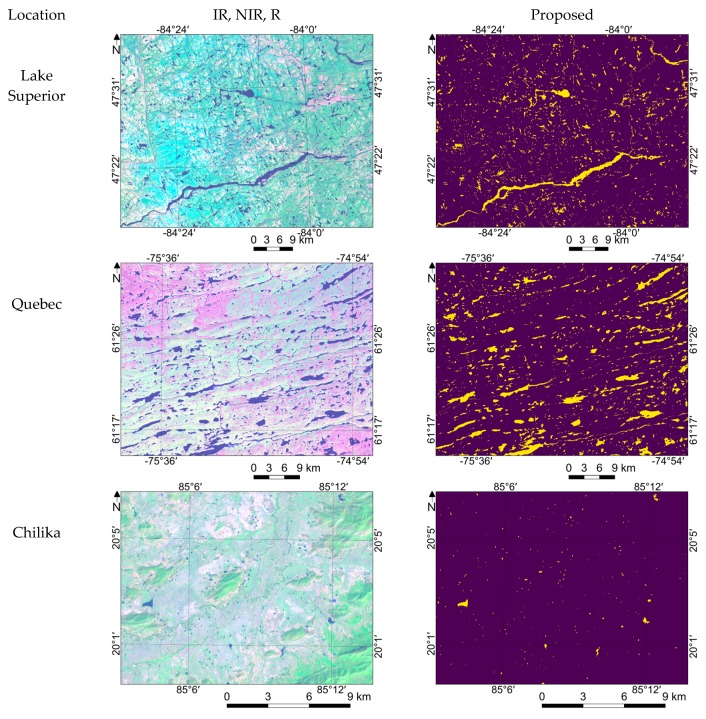

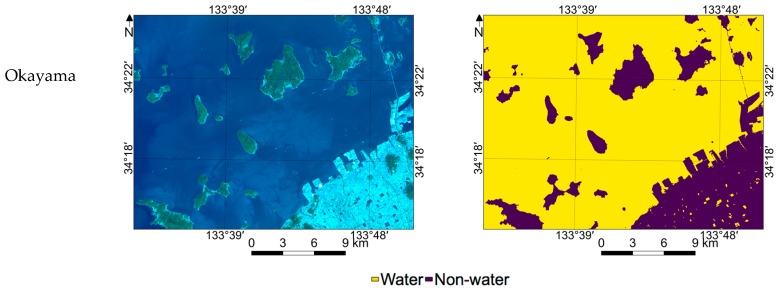
Demonstration of results in detecting smaller water and non-water features.

Figure 7 shows four various examples from parts of Lake Superior, Quebec, Okayama, and Chilika. For Lake Superior and Quebec, small lakes and narrow streams were detected. In the case of Quebec, small water bodies observed in the summer due to ice melting and water bodies surrounded by snow/ice regions were also estimated accurately, where several water region estimation algorithms struggle to estimate this correctly. In the case of Chilika, very small water bodies such as ponds which may be used for agricultural purposes were also detected. In the case of Okayama, apart from very small water bodies surrounded by buildings, small non-water features such as small islands also were estimated accurately. Moreover, very small non-water features like ships and bridges (Great Seto Bridge) were also detected. The given examples demonstrate the feasibility of the proposed model in estimating smaller water and non-water features, too. Also check Appendix B for more examples for water/non-water region estimation at various locations. A qualitative comparison carried out with Pekel et al. [13]) also shown in Appendix B.

### 3.5. Preliminary Results of Global Water Survey

In order to demonstrate the global adaptability of the proposed method, we processed 900 Landsat-8 images captured over East Asia and India for the year of 2017. The characteristics of the system used for global water region estimation are as follows: AWS Region: Oregon, EC2 Instance Type: m4.2xlarge (8core, 32GB memory,) OS: CentOS7, UI: based on Land Browser [55] 

**Figure 8:**
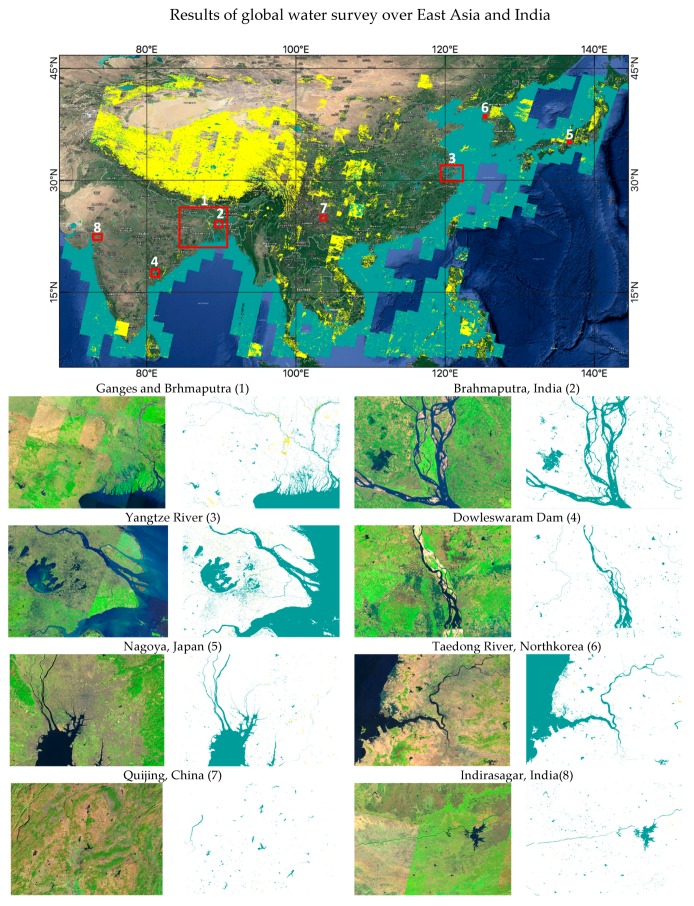
Demonstration of water region estimation using 900 Landsat-8 images over East Asia and India (green: water; yellow: cloud).

Figure 8 shows the water region estimation over East Asia and India. The green color represents estimated water regions and the yellow color represents the clouds which were estimated using Fmask [50,51]. Cloud-covered regions were not used for water estimation. Figure 8 also demonstrates eight various water features from small to large sizes using the denoted bounding boxes. Each bounding box region were projected to highlight the estimated water regions clearly with higher spatial resolution for visualization purposes. Large river basins, deltas, reservoirs, dams, and other small water bodies from various places are shown.

### 3.6. Performance Evaluation of the Proposed Method for Sentinel-2 Images

The proposed method was basically developed for Landsat-8 images; however, in order to evaluate the performance for other satellite images which have similar spectral band characteristics, we also tested randomly selected Sentinel-2 [56] images. The blue (0.490 μm), green (0.560 μm), red (0.665 μm), NIR (0.842 μm), SWIR1 (1.610 μm), and SWIR2 (2.190 μm) bands of Sentinel-2 were used to generate five features (*x*_1_, *x*_2_, ..., *x*_5_) like in the case of Landsat-8. Further, the proposed PDWF parameters were used to estimate water/non-water regions in the case of cloud-free, sunglint-free, and snow-free Sentinel-2 images. 

**Figure 9:**
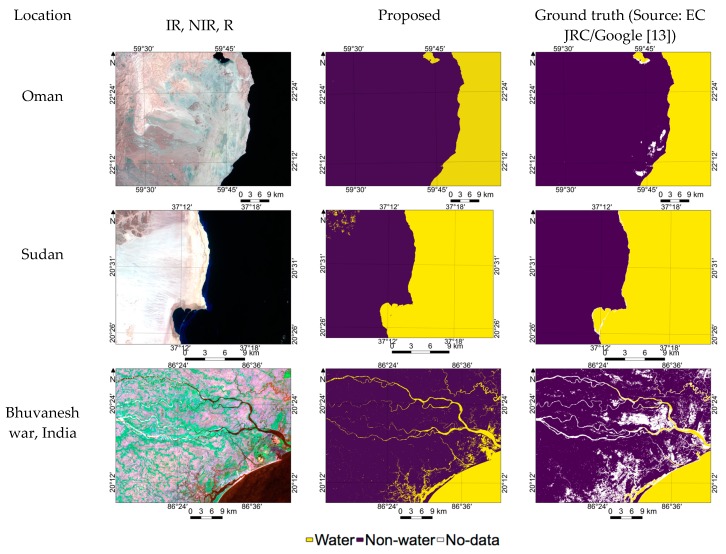
Demonstration of water region estimation using Sentinel-2 images over randomly selected locations.

In case of sunglint-affected images, a sunglint correction algorithm was used the same as for Landsat-8 images. Thee randomly selected Sentinel-2 level-1c data were used for quantitative and qualitative evaluation. Table 7 shows that in all the three locations, the proposed method provides reliable results with acceptable accuracy. Figure 9 shows that in the case of Sudan, FPs are observed in the hill shadow region. In all the locations, higher commission error (0.0116) compared to omission error occurred due to higher FPs. This can be managed by fine-tuning the PDWF parameters using a limited number of Sentinel-2 images with corresponding ground truths. However, in general, Figure 9 and Table 7 show that the proposed method with PDWF parameters is also applicable for water/non-water region estimation using Sentinel-2 images.

## 4. Conclusions

Water region estimation is considered one of the fundamental remote sensing applications. This study aimed to develop an efficient and globally adaptable water region estimation method. The main challenge was to develop a method which uses only Landsat-8 spectral bands without any additional datasets. Global adaptability of the parameters and threshold for water/non-water region estimation is another challenging task. Therefore, this study investigated perceptron learning to address these issues by developing a formula (PDWF) using spectral bands only. PDWF was focused on reducing FPs due to hill shadows, building shadows, and dark soils. We showed that PDWF succeeded in water/non-water region estimation from clear Landsat-8 images. In the case of sunglint-affected Landsat-8 images, we required sunglint correction. This study defined a sunglint correction algorithm which adjusts the index values for each pixel according to the *SA*. This sunglint correction was able to reduce the number of FNs induced by sunglint. A snow/ice correction algorithm was used for snow/ice-covered Landsat-8 images. Hence, an automated approach can be used for water/non-water region estimation by combining PDWF, sunglint correction, and snow/ice correction algorithms.

Five-fold model training was used to derive the best parameters because weight initialization is crucial for perceptron learning. The performance of the PDWF was evaluated using various case studies and compared with the results of index-based approaches (MNDWI and AWEI) and a deep learning model (U-net). The evaluations show that PDWF outperforms all other methods in terms of quantitative and qualitative analysis. PDWF was able to distinguish water from low-spectral-response features such as hill shadows, building shadows, and dark soils, whereas the other methods failed to do so. In all case studies, improved water/non-water region estimation using PDWF significantly reduced the number of FPs and FNs. We compared the performance in various types of locations such as urban areas, hilly areas, and low-lying coastal areas. We also qualitatively evaluated the performance of the proposed method in detecting smaller water/non-water features. Very small water/non-water features were successfully detected in several selected locations. A resource and processing time evaluation of PDWF and U-net showed that PDWF was very much faster compared to U-net in both CPU and GPU cases. Moreover, compared to deep learning black box approaches, PDWF can explained theoretically. Of course, it is possible to fine tune U-Net or find a deep learning model that can perform best in global scale; however, we would like to emphasize that simplicity of the perceptron model investigated in this work is worth to be noted as an option regarding demonstrated performance and computational efficiency on large-scale satellite data. 

The PDWF was developed using TOA reflectance data which were not atmospherically corrected; hence, it can easily be adapted to any region since atmospheric correction is not necessary. The preliminary results of our global water survey system are quite promising, and one of our future goals is to develop a Landsat-8-based global temporal water change detection system which can be used for various applications. We also hope to generate a new set of PDWF parameters for Sentinel-2 using transfer learning from the current Landsat-8 PDWF parameters. This can help to produce more frequent water surveys on a global scale.

## Figures and Tables

**Table 1:** Landsat-8 data used for training, validation, and testing.

Usage	Location	Date
Training and validation	Gulf of California, Mexico	2017-01-22
	Red Sea, Saudi Arabia	2015-11-01
	Numbulwar, Australia	2017-08-08
	Christchurch, New Zealand	2015-10-10
	Mansel island, Canada	2016-08-13
	Abu Dhabi, UAE	2017-11-25
	Surat, India	2017-11-14
	Hanover, Germany	2017-03-28
	Yancheng, Chaina	2016-02-18
	East Siberian sea, Russia	2015-09-05
	Rio de Janeiro, Brazil	2017-09-14
	Yenisei river, Russia	2017-07-21
	Nitchequon, Canada	2016-09-27
	Chita, Russia	2017-09-11
	Hong Kong	2016-02-07
Test images with clear observation	Lake Malawi, Africa	2016-06-16
	Great barrier reef, Australia	2016-08-14
	Chilika lake, India	2017-02-07
	Chiba, Japan	2017-03-20
	Lake Vi­ctoria, Africa	2016-08-17
Test images with sunglint and snow/ice	Gulf of California, Mexico	2017-08-02
	Red Sea, Saudi Arabia	2017-05-14
	Yamamoto, Japan	2015-05-02

**Table 2:** Five-fold cross model training, validation, and testing results.

Folds	Training Accuracy			Validation Accuracy			Testing Accuracy			Overall Accuracy		
	Feature (Pretrained)	Feature (Random)	Bands (Random)	Feature (Pretrained)	Feature (Random)	Bands (Random)	Feature (Pretrained)	Feature (Random)	Bands (Random)	Feature (Pretrained)	Feature (Random)	Bands (Random)
1	0.993	0.549	0.548	0.997	0.734	0.661	0.997	0.717	0.716	0.996	0.666	0.642
2	0.995	0.572	0.540	0.990	0.658	0.707	0.997	0.718	0.716	0.994	0.649	0.654
3	0.997	0.593	0.589	0.994	0.580	0.580	0.999	0.703	0.874	0.997	0.625	0.685
4	0.993	0.556	0.992	0.996:	0.709	0.993	0.996	0.717	0.998	0.994	0.661	0.994
5	0.984	0.996	0.572	0.987	0.995	0.661	0.987	0.998	0.715	0.986	0.996	0.649

**Table 3:** Quantitative evaluation of the performance of Automatic Water Extraction Index (AWEI), MNDWI, U-net, and Perceptron-Derived Water Formula (PDWF).

Location	Method	FPs + FNs	Commission Error	Omission Error	Accuracy
Lake Malawi	AWEI (Feyisa et al. [14])	29,391	0.0001	0.0022	0.9992
	MNDWI (Xu et al. [22])	59,668	0.0037	0.0008	0.9985
	U-net (Olaf et al. [33])	8937	0.0001	0.0012	0.9997
	PDWF	23,327	0.0001	0.0016	0.9994
Great Barrier Reef	AWEI (Feyisa et al. [14])	491,210	0.0013	0.0557	0.9853
	MNDWI (Xu et al. [22])	356,618	0.0382	0.0001	0.9895
	U-net (Olaf et al. [33])	16,032	0.0003	0.0003	0.9995
	PDWF	16,219	0.0015	0.0002	0.9995
Chilika Lake	AWEI (Feyisa et al. [14])	31,180	0.0041	0.0030	0.9991
	MNDWI (Xu et al. [22])	322,692	0.0648	0.0016	0.9917
	U-net (Olaf et al. [33])	42,869	0.0056	0.0039	0.9989
	PDWF	29,565	0.0033	0.0031	0.9992
Chiba	AWEI (Feyisa et al. [14])	413,482	0.0018	0.0441	0.9843
	MNDWI (Xu et al. [22])	1,199,664	0.1123	0.0001	0.9556
	U-net (Olaf et al. [33])	48,028	0.0032	0.0009	0.9982
	PDWF	29,685	0.0021	0.0009	0.9989
Lake Victoria	AWEI (Feyisa et al. [14])	12,814	0.0004	0.0003	0.9996
	MNDWI (Xu et al. [22])	26,905	0.0017	0.0001	0.9993
	U-net (Olaf et al. [33])	37,541	0.0032	0.0002	0.9990
	PDWF	10,982	0.0004	0.0002	0.9997
Average	AWEI (Feyisa et al. [14])	195,615	0.0015	0.0210	0.9935
	MNDWI (Xu et al. [22])	393,109	0.0441	0.0005	0.9869
	U-net (Olaf et al. [33])	30,681	0.0025	0.0013	0.9991
	PDWF	21,955	0.0015	0.0012	0.9993

**Table 4:** Quantitative evaluation of the performance of AWEI, MNDWI, U-net, and PDWF in the case of sunglint-affected and snow/ice-covered images.

Location	Method	FPs + FNs	Commission Error	Omission Error	Accuracy
Red Sea (sunglint)	AWEI (Feyisa et al. [14])	9,424,695	0.0035	0.8271	0.7651
	MNDWI (Xu et al. [22])	176,670	0.0148	0.0001	0.9956
	U-net (Olaf et al. [33])	396,929	0.0106	0.0028	0.9903
	Proposed	717,487	0.0001	0.0614	0.9823
Gulf of California (sunglint)	AWEI (Feyisa et al. [14])	13,181,792	0.0006	0.4765	0.7602
	MNDWI (Xu et al. [22])	86,539	0.0030	0.0001	0.9977
	U-net (Olaf et al. [32])	3,525,161	0.2190	0.0415	0.9114
	Proposed	3,267,434	0.0003	0.1163	0.9167
Yamamoto	AWEI (Feyisa et al. [14])	4,467,484	0.4754	0.3363	0.8677
	MNDWI (Xu et al. [22])	3,303,202	0.3613	0.0003	0.9026
	U-net (Olaf et al. [32])	105,065	0.0035	0.0001	0.9969
	Proposed	269,177	0.0374	0.0074	0.9920
Average	AWEI (Feyisa et al. [14])	9,024,657	0.1598	0.5466	0.7977
	MNDWI (Xu et al. [22])	1,188,803	0.1264	0.0001	0.9653
	U-net (Olaf et al. [32])	1,342,385	0.0777	0.0148	0.9662
	Proposed	1,418,032	0.0126	0.0617	0.9637

**Table 5:** Comparison in terms of processing time for prediction.

Methods	Number of Parameters	Time with only CPU			Time with GPU		
		Create Patches (128)	Prediction	Back to Original Shape	Create Patches (128)	Prediction	Back to Original Shape
U-net (Olaf et al. [32])	7,766,915	8.35	1300.52	27.50	3.45	47.45	10.34
PDWF	12	N/A	2.66	N/A	N/A	0.06	N/A

**Table 6:** Quantitative evaluation of the performance of the proposed method and improvements after sunglint and snow/ice correction.

Location	Correction	FPs + FNs	Commission Error	Omission Error	Accuracy
Gulf of California	Before sunglint correction	3,267,434	0.0003	0.1163	0.9167
	After sunglint correction	21,697	0.0007	0.0001	0.9994
	After snow/ice correction	21,697	0.0007	0.0001	0.9994
Red Sea	Before sunglint correction	717,487	0.0001	0.0614	0.9823
	After sunglint correction	51,981	0.0039	0.0004	0.9987
	After snow/ice correction	51,981	0.0039	0.0004	0.9987
Yamamoto	Before sunglint correction	269,177	0.0374	0.0074	0.9920
	After sunglint correction	268,633	0.0428	0.0013	0.9921
	After snow/ice correction	62,666	0.0092	0.0014	0.9981
Average	Before sunglint correction	1,418,032	0.0126	0.0617	0.9637
	After sunglint correction	114,103	0.0158	0.0006	0.9967
	After snow/ice correction	45,448	0.0046	0.0006	0.9988

**Table 7:** Quantitative evaluation of the performance of the proposed method in the case of Sentinel-2 images.

Location	Date	FPs + FNs	Commission Error	Omission Error	Accuracy
Oman	2018-09-20	442,194	0.0116	0.0001	0.9963
Sudan	2018-09-27	670,097	0.0073	0.0004	0.9931
Bhuvaneshwar	2018-01-05	606,218	0.0159	0.0016	0.9928
Average		572,836	0.0116	0.0007	0.9940

**Figure B1:**
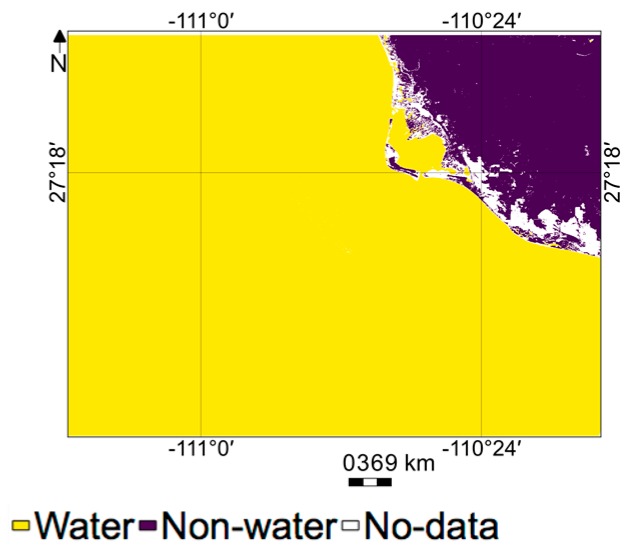
An example of a ground truth from Gulf of California region.

**Figure B2:**
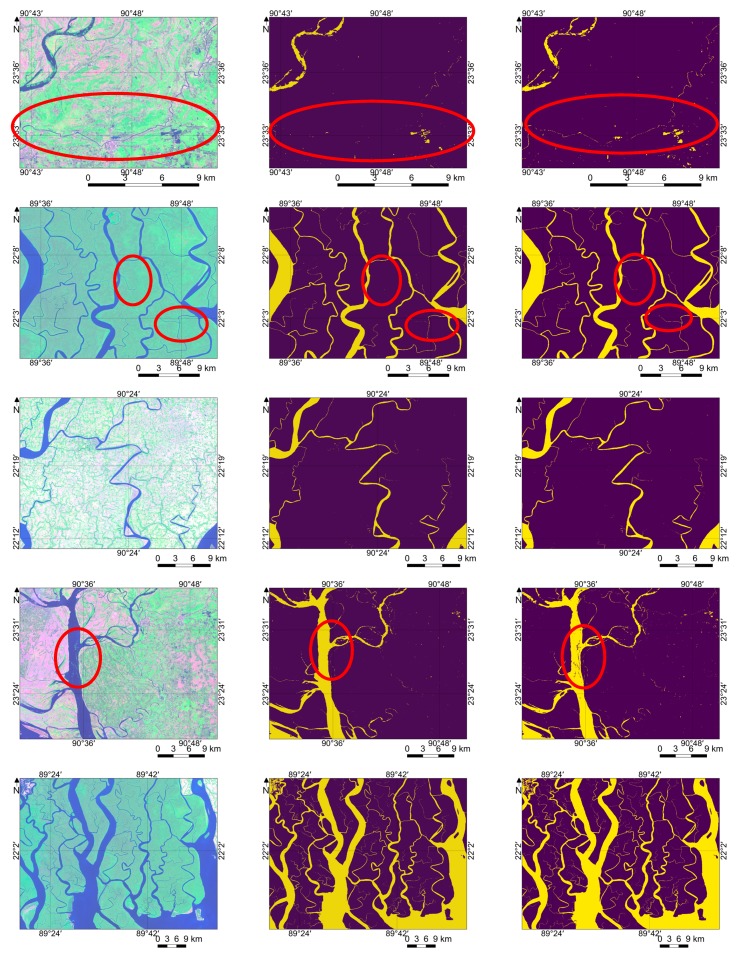
Demonstration of water/non-water region estimation results derived from Pekel et al. [13] and proposed method.

**Figure B3:**
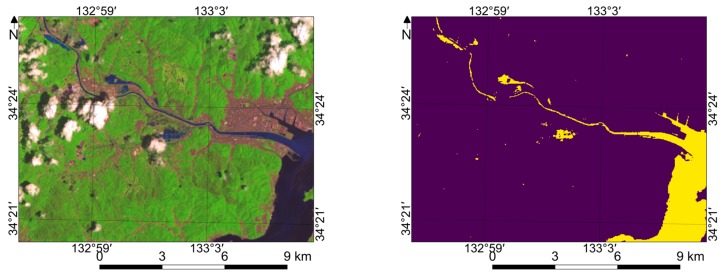
Flood-inundated muddy water extracted during the Hiroshima flood, July 2018.

**Figure B4:**
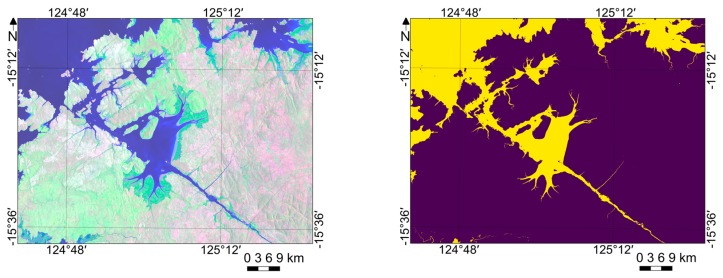
Highly complicated coastal region of Kimberley, Australia.

**Figure B5:**
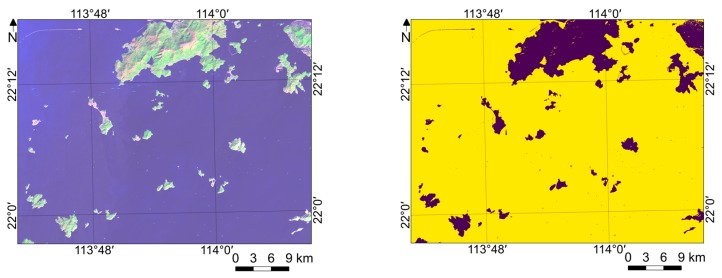
Islands and other coastal water/non-water features detected in Hong Kong.

**Figure B6:**
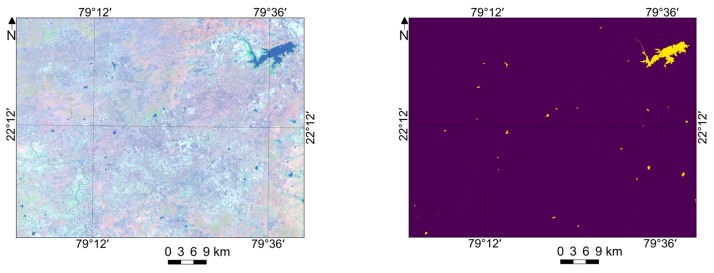
Water/non-water region estimation using Landsat-8 image collected on 2014-04-16.

**Figure B7:**
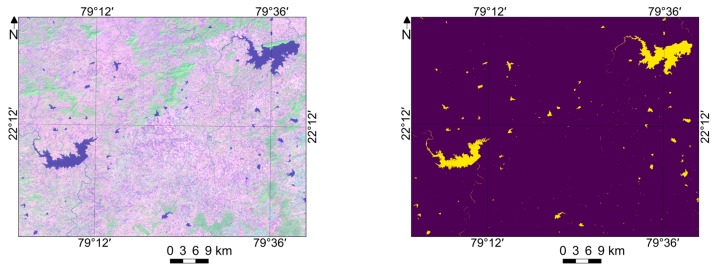
Water/non-water region estimation using Landsat-8 image collected on 2016-10-30.

## Appendix A

NDWI = (Green − NIR)/(Green + NIR)

MNDWI = (Green – SWIR1)/(Green + SWIR1)

AWEInsh = 4 × (Green − SWIR1) − (0.25 × NIR + 2.75 × SWIR2)

AWEIsh = Blue + 2.5 × Green − 1.5 × (NIR + SWIR1) − 0.25 × SWIR2

Previous formula [22] = 2 × (Green − NIR) + (Blue − NIR) + (Red − SWIR) − ((1.25 × SWIR1) + (0.5 × SWIR2))

Thermal band data can be converted from spectral radiance to top of atmosphere brightness temperature using the thermal constants in the MTL file.

Conversion to Top of Atmosphere Brightness Temperature:

BT = (k2/(log(k1/(Ml_rad × {Band10} + Ad_rad) + 1))) − 273.15

where BT is the top of atmosphere brightness temperature, K1 is the band-specific thermal conversion constant from the metadata, and K2 is the band-specific thermal conversion constant from the metadata.

## Appendix B

The ground truth images that we used for training and evaluation also includes "no-data" labels where the class-label is unknown.. In a GSW (Pekel et al. [13]) water occurrence map, the pixels which we were not able to distinguish manually as water or non-water were considered as no-data. An example of the ground truth is shown in Figure B1 (Source: EC JRC/Google [13]).

In order to investigate the feasibility of the proposed method, this study qualitatively compared our results with those of GSW (Pekel et al. [13]) by visual inspection (Figure B2). GSW (Pekel et al. [13]) used an expert system classifier which includes several additional datasets such as DEM, glacier data, urban area data, volcanic lava data, etc., while this study did not use any additional data. However, the visual comparison over selected regions shows that the proposed method is slightly better; of course, there might be some performance differences quantitatively if tested and compared for large-scale data. Several small water features demarcated in the Figure B2 below were detected using the proposed method while the method by Pekel et al. [13] failed to do so. Water/non-water region estimation were carried out for several images other than those shown in Table 1. This section intended to demonstrate the feasibility of the proposed method for water/non-water region estimation in various parts of the world under various conditions.

Figure B3, Figure B4 and Figure B5 demonstrate water/non-water region estimation in various locations and situations.

Figure B6 and Figure B7 show the temporal changes of water/non-water region in Machagora Dam, Madhya Pradesh region.
